# Biogenesis of Developmental Master Regulatory 27nt-RNAs in *Stylonychia*—Can Coding RNA Turn into Non-Coding?

**DOI:** 10.3390/genes10110940

**Published:** 2019-11-18

**Authors:** Jan Postberg, Patrick Philipp Weil, Anton Pembaur

**Affiliations:** Clinical Molecular Genetics and Epigenetics, Faculty of Health, Centre for Biomedical Education & Research (ZBAF), Helios University Hospital Wuppertal (Zentrum für Forschung in der Klinischen Medizin—ZFKM), Witten/Herdecke University, Alfred-Herrhausen-Str. 50, 58448 Witten, Germany; patrick.weil@uni-wh.de (P.P.W.); anton.pembaur@uni-wh.de (A.P.)

**Keywords:** macronuclear development, RNA-induced DNA replication interference, ciliates, nuclear dualism

## Abstract

In the ciliate *Stylonychia,* somatic macronuclei differentiate from germline micronuclei during sexual reproduction, accompanied by developmental sequence reduction. Concomitantly, over 95% of micronuclear sequences adopt a heterochromatin structure characterized by the histone variant H3.4 and H3K27me3. RNAi-related genes and histone variants dominate the list of developmentally expressed genes. Simultaneously, 27nt-ncRNAs that match sequences retained in new macronuclei are synthesized and bound by PIWI1. Recently, we proposed a mechanistic model for ‘RNA-induced DNA replication interference’ (RIRI): during polytene chromosome formation PIWI1/27nt-RNA-complexes target macronucleus-destined sequences (MDS) by base-pairing and temporarily cause locally stalled replication. At polytene chromosomal segments with ongoing replication, H3.4K27me3-nucleosomes become selectively deposited, thus dictating the prospective heterochromatin structure of these areas. Consequently, these micronucleus-specific sequences become degraded, whereas 27nt-RNA-covered sites remain protected. However, the biogenesis of the 27nt-RNAs remains unclear. It was proposed earlier that in stichotrichous ciliates 27nt-RNA precursors could derive from telomere-primed bidirectional transcription of nanochromosomes and subsequent Dicer-like (DCL) activity. As a minimalistic explanation, we propose here that the 27nt-RNA precursor could rather be mRNA or pre-mRNA and that the transition of coding RNA from parental macronuclei to non-coding RNAs, which act in premature developing macronuclei, could involve RNA-dependent RNA polymerase (RDRP) activity creating dsRNA intermediates prior to a DCL-dependent pathway. Interestingly, by such mechanism the partition of a parental somatic genome and possibly also the specific nanochromosome copy numbers could be vertically transmitted to the differentiating nuclei of the offspring.

## 1. Introduction

Many small non-coding RNAs (ncRNAs) inhabit control function for cellular processes including the regulation of gene expression and chromatin structure. The RNA interference (RNAi)-dependent formation of heterochromatin might be best understood in fission yeast [1], *Arabidopsis* [2] and the ciliate *Tetrahymena* (class: Oligohymenophorea) [3,4], wherein extensive developmental chromatin reorganization processes occur. Single ciliate cells contain two types of nuclei, solitary or multiple germline micronuclei and somatic macronuclei. A macronucleus develops from a micronucleus-derivative during sexual reproduction, which starts when two cells of different mating types conjugate [5,6]. Macronuclear development in the course of ciliate sexual reproduction involves chromatin reorganization and programmed DNA elimination. Whereas this process is a major differentiator of the Ciliophora taxon within in the tree of eukaryotic life, evolutionary history resulted in several fundamentally different variations on the molecular mechanisms and how these peculiar single-celled organisms keep control over these sophisticated genome rearrangements. In this mini-review, we address the open problem: how development-specific small ncRNAs could become synthesized in spirotrichous ciliates like *Stylonychia* or *Oxytricha*, since this key mechanism seems to differ fundamentally from *Tetrahymena* or *Paramecium*—two genera for which much more data is available to date.

## 2. Small ncRNA Biogenesis in *Tetrahymena* and *Paramecium* Involves Non-Coding RNA Precursors Transcribed in the Germline Micronucleus 

In *Tetrahymena*, non-coding ‘scnRNA’ guide the programmed elimination of germline-restricted DNA sequences, which become targeted by sequence homology. Their precursors are bi-directionally transcribed from the micronuclear chromosomes encompassing germline-restricted and macronucleus-destined sequences. Then, Dicer-like activity by Dcl1p is required for the production of ~29nt-RNAs, which become bound by the PIWI-homolog Twi1p followed by slicing, i.e. the passenger strand removal, and 3’-end 2’-*O*-methylation via the methyltransferase Hen1p. Subsequently, a genome comparison/selection process (‘scanning’) is undergone in the degrading parental macronucleus, leading to the degradation of ~29nt-RNAs matching to homologous macronucleus sequences. The resulting scnRNAs target micronucleus-specific sequences not required in the mature macronucleus. Subsequently, H3K9me3 and H3K27me3 become enriched at these sequences. This process involves the activity of the histone lysine methyltransferase (KMT) EZL1. Prior to their elimination, these sequences become transformed into heterochromatin through binding of the chromodomain protein PDD1p at both H3K9me3 and H3K27me3 [3,4]. A similar ‘genome-scanning model’ is proposed for *Paramecium*. Here, ~25nt-RNAs result from precursors, which derive from whole micronuclear genome transcription. In the parental macronucleus, they undergo a genome comparison against macronuclear non-coding transcripts, which are constitutively produced in vegetative cells [7].

## 3. Developmental Small Non-Coding RNAs Do Not Target Micronucleus-Specific Sequences in Spirotrichous Ciliates, but Safeguard the Retention of Macronucleus-Destined Sequences 

Interestingly, in ciliates belonging to the class Spirotrichea an apparently inverse role for developmental small non-coding RNAs was observed. Here, 27nt-RNAs are synthesized from the parental macronucleus. The macronulceus genome in *Oxytricha trifallax* and *Stylonychia lemnae* comprises of so-called nanochromosomes harbouring mostly one or few genes flanked by discrete telomeric repeats. In a mature macronucleus, each nanochromosome occurs in a specific copy number [8,9]. In both species, developmental 27nt-RNAs target macronucleus-specific sequences in developing macronuclei in association with Argonaute/PIWI-protein homologs [10,11,12,13]. For *Oxytricha* it was proposed that these 27nt-RNAs protect specific sequences from being degraded. However, no deeper mechanistic insight was provided [10]. A valuable study complemented this study by a biochemical characterization of the *Oxytricha* 27nt-RNAs demonstrating that they are not modified by 2’-*O*-methylation at their 3’-end, in contrast to *Tetrahymena* scnRNAs [14]. Outgoing from the observation that the vast majority of 27nt-RNAs match macronuclear nanochromosomes bi-directionally but omit their telomeres, the same study hypothesizes that the biogenesis of 27nt-RNA precursors could originate from telomere-primed transcription of both DNA strands. Theoretically, this is a reasonable hypothesis, whose mechanism would safeguard the even transformation of the macronuclear DNA sequence information (with the exception of telomeres, which become de novo added by telomerase) and possibly also the nanochromosome copy numbers to a long non-coding RNA level. However, it is challenged by the observation that telomeres occur in a very stable G-quadruplex conformation that most probably is not easily accessible for telomere-priming activity [15,16]. To date in *Stylonychia*, a telomeric G-quadruplex resolving RecQ-like helicase activity (in association with telomerase) was exclusively observed in the replication band of vegetative macronuclei, but not in the parental macronulcear fragments during sexual reproduction [17]. Therefore, we believe that our data from *Stylonychia* justifies an alternative hypothesis how the biogenesis of 27nt-RNAs could occur. Our thoughts will be exemplified in the following paragraphs.

## 4. Developmental 27nt-RNAs in *Stylonychia* and Their Potential Role as Heterochromatization Preventers at Macronucleus-Destined Sequences

**Figure 1:**
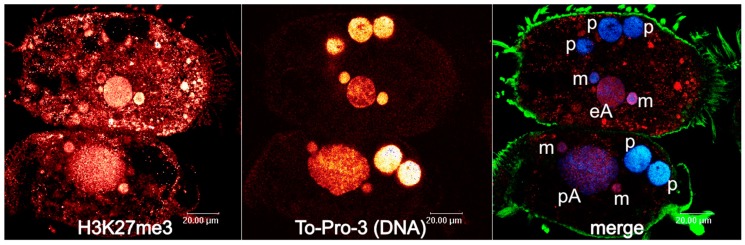
Two *Stylonychia* cells with developing macronuclei at an earlier stage of chromosome polytenization (upper cell) and at a stage of maximum polytenization (cell below). Left: H3K27me3; centre: To-Pro-3 counterstaining of DNA; right: merge (red: H3K27me3, blue: DNA, green: alpha-tubulin). H3K27me3 exhibits strong signals in micronuclei and developing macronuclei in both successive developmental stages, whereas cytoplasmic enrichment is more obvious throughout the earlier stages of chromosome polytenization. This might be indicative of cytoplasmic post-translational histone H3K27me3 modification taking place prior to its specific chromatin deposition. Abbreviations: p: parental macronuclear fragment; m: micronucleus; M: macronucleus; eA: early macronuclear anlage; pA: polytene macronuclear anlage.

*Stylonychia* has a long history as a model for macronuclear differentiation. Here, developmental chromatin reorganization eventually leads to the formation of >16,000 different gene-sized linear nanochromosomes in the mature macronucleus [18], whereby over 95% of the micronuclear sequences become degraded, most of which comprise repetitive and unique sequences from micronucleus-specific intergenic DNA [5]. Apart from this bulk DNA, internal eliminated sequences (IESs) interrupt macronucleus-destined sequences (MDSs) within many micronuclear genes. MDSs frequently occur in scrambled disorder, when compared with their proper arrangement in mature nanochromosomes [19]. IESs can be as short as 10 bp and must be removed during macronuclear differentiation [20]. Prior to the reduction of germline-specific sequences the diploid zygote genome undergoes polytene chromosome formation in a first phase of serial DNA replication [21]. In this stage, IES excision and reordering of MDSs in scrambled genes take place [22], prior to the massive reduction of bulk DNA sequences, which eventually leads to the breakdown of polytene chromosomes [5,6]. In *Stylonychia* histone post-translational modifications (PTM) have been extensively studied [12,23,24]. For example, H3K27me3 accumulates at micronucleus-specific sequences prior to their elimination, and ultrastructural studies show that excised DNA occurs in form of condensed chromatin [25]. Macronuclear development depends on sncRNAs [12] and the Argonaute family protein PIWI1, which appears to be a driver for RNA trafficking and trans-nuclear crosstalk [11,24]. Furthermore, a not yet deeply characterized RNA species might be involved in MDS reordering, IES excision, nanochromosome copy number determination and telomere addition [8,9,26,27]. The most recent studies suggest that in *Stylonychia* the deposition of multiple histone variant-containing nucleosomes into chromatin of different nuclear types and their association with specific sequence-classes play a superior role in developmental chromatin reorganization with *Stylonychia* being among the list of eukaryotes encoding very high numbers of histone variants [23]: six H2A, four H2B, nine H3 and two H4. Due to discrete amino acid substitutions, gains or losses it seems clear that the range of possible PTMs for each of the 9 histone H3 variants but also other histone types is diversified. We showed that the spatiotemporal occurrence of histone variants in the life cycle of *Stylonychia* is highly regulated, and we found evidence that the expression of some H3 variants might be influenced by PIWI1-dependent RNAi [23]. More recent findings, moreover, highlight the possibility that non-coding 27nt-RNAs could directly play a crucial role in the regulation of histone variant deposition into polytene chromosomes of developing macronuclei: The ‘RNA-induced DNA replication interference’ (RIRI)-model provides a mechanistic explanation how 27nt-RNAs could protect DNA from being lost during macronuclear development [13]. Accordingly, PIWI1/27nt-RNA complexes block polytenization of covered MDSs, thus limiting the replication-dependent de novo deposition of nucleosomes. Meanwhile, a histone H3 variant (H3.4) that is permissive for H3K27me3 becomes enriched at specific sites under ongoing replication, whereas MDSs are omitted from the deposition of H3K27me3. Here, it appears that trimethylation of lysine 27 takes place on cytoplasmic H3 before its site-specific chromatin deposition in the developing macronucleus (Figure 1). The observation of cytoplasmic post-translational histone modification seems unusual, but co-translational lysine methylation of H3 was recently observed also in human cervical cancer cells (HeLa) [28]. This important observation is supportive for the RIRI-model, since the requirement of a putative site directed H3K27-specific histone methyltransferase activity would be obsolete. Eventually, this tight spatiotemporal coordination of histone-variant deposition leads to the establishment of a chromatin structure barcode, where the formation of heterochromatin bands via H3.4K27me3 is limited to those regions not protected by PIWI1/27nt-RNA complexes. Consequently, bulk intergenic DNA is specified for elimination and thus becomes functionally separated from sequences that must be retained in mature macronuclei.

## 5. A Minimalistic Model for the Biogenesis of 27nt-RNAs—Is mRNA/pre-mRNA the Substrate?

It is obvious that in spirotrichous ciliates 27nt-RNAs are developmental key regulators targeting specific DNA sequences and influencing local chromatin structures. In search of the vanguard master regulatory process that leads to the de novo mobilization of these non-coding 27nt-RNAs during macronuclear development, we followed the rationale of minimalism. Thereafter, a basic biological process with known molecular actors could provide a more plausible hypothesis for a 27nt-RNA biogenesis mechanism than a theoretical mechanism that would require the taxon-specific evolution of multiple unrecognized molecular factors. In this context, we extract earlier and novel relevant observations, which enable us to shape the hypothesis that the substrate for the biogenesis of developmental non-coding RNAs could be the pool of coding mRNAs that become transcribed in the parental (old) macronucleus. 

**Figure 2:**
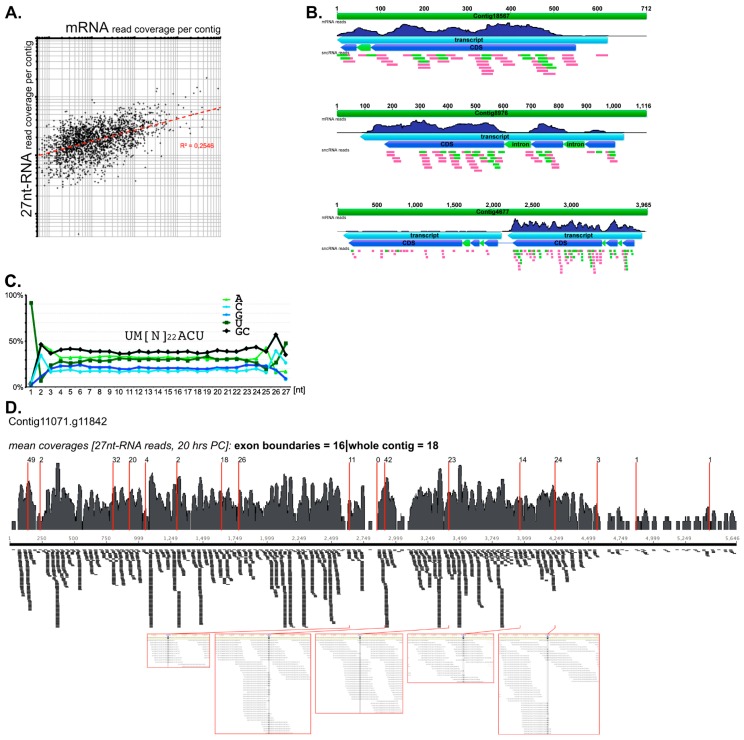
(**a**) A correlation plot demonstrates the positive correlation of normalized read coverages for 27nt-RNAs and mRNAs with respect to 2131 genes. (**b**) (modified after [13])**.** Three exemplary nanochromosome contigs with illustrations of mapped reads for mRNAs (transcript, dark blue track) and 27nt-RNAs (pink bars) as well as 21–22nt-RNAs (light green bars). Interestingly, some 27/21–22nt-RNAs map to introns (green arrows interrupting coding sequence, CDSs). Importantly, the contig at the bottom of the illustrations is an example for a nanochromosome that contains 2 genes, which become transcribed at different levels. The numbers of mapping 27/21–22nt-RNAs is much higher for the more transcribed gene, strongly supporting the direct correlation between mRNA quantities and 27/21–22nt-RNA quantities. (**c**) (modified after [13])**.** Nucleotide composition of 27nt-RNAs (A/U green; G/C blue): Position 1 is almost invariantly uridine (U; >90%) followed by adenosine (A) or cytidine (C) (>80%) and at position 3 frequently A (~40%) occurs. The guanosine (G):C and A:U ratios between positions 3 to 24 are balanced, suggesting a double stranded precursor. At the 3’-end position 25 is frequently A (>40%), position 26 C or U (>65%) and position 27 is often U (>45%). The 21–22nt-RNA-pattern is similar, but not identical to 27nt-RNAs [13]. (**d**) Mapping of 27nt-RNA reads to exon boundaries of Contig11071.g11842, a CDS contig with high 27nt-RNA read coverage. This contig consists of 18 exons and 17 exon boundaries, respectively. Exon boundaries are marked by red lines. Below, selected magnifications are shown wherein the framed columns mark the position of the exon boundaries. These illustrations demonstrate the central arrangement of several exon boundaries with respect to the position of numerous mapped 27nt-RNA reads (sequence information not visible at the selected level of resolution).

In *Stylonychia*, deep sequencing revealed two different fractions of small RNAs, which are 21–22 nt and 27 nt in size, respectively [13], which is reminiscent of *Oxytricha* [10,14]. Whereas the 21–22nt-RNAs seem to be present throughout vegetative growth and the sexual reproduction of *Stylonychia* an occurrence of 27nt-RNAs over baseline is not observable during vegetative growth. 27nt-RNAs emerge at the onset of conjugation between mating cells. Shortly afterwards a massive, temporally restricted 27nt-RNA enrichment takes place, which is reminiscent of a selective 27nt-RNA amplification activity, possibly by a putative RDRP-activity. Interestingly, three nanochromosomes containing RDRP candidate genes were identified in *Styloynchia* [13]. Previously, we have speculated marginally that in *Stylonychia* the substrate for the biogenesis of 27nt-RNAs (and 21–22nt-RNAs) could be mRNA [13]. A reinvestigation of RNA-seq datasets using 2,131 contigs for mapping of small non-coding RNA reads and mRNA reads confirms that there is a positive correlation between the read counts per contig between both 27nt-RNAs and mRNA (Figure 2A). The inspection of multiple annotated nanochromosomal contigs revealed more evidence for a direct link between the quantity levels of mRNA and 27nt-RNAs. Figure 2B is a modified depiction of those previous analyses [13], which shows three exemplary nanochromosomal contigs being representative with respect to the following observations: 1. Transcribed mRNA coverage fits well with the previous genome annotation [18], whereas we could not observe mapped 27nt-RNA reads associated with non-transcribed sequences. 2. There is a remarkable overlap between the mRNA read coverage and the 27nt-RNA coverage. Strikingly, contig4677 (Figure 2B bottom) contains two transcribed genes, whereby both transcripts occur in very different quantities. Here, many more 27nt-RNA reads mapped to that highly expressed gene. These observations suggest that a pool of transcribed mRNAs could be the substrate for 27nt-RNA biogenesis. Interestingly, we observed that 27nt-RNAs occasionally mapped to annotated introns. This might be indicative that mature mRNAs but also non-edited pre-mRNAs could serve as source material. Moreover, the nucleotide composition of 27nt-RNAs is pointing at a possible upstream dsRNA precursor: For *Stylonychia* 27nt-RNAs between nucleotides 3–24 there are almost equal ratios of A/U or G/C, respectively (Figure 2C). Exclusively at the 5’- and 3’-ends we recognized a certain degree of sequence conservation, which might be indicative for a recognition motif (27nt-RNA consensus: 5’-UM[N]_22_ACU-3’) (modified after [13]). Interestingly, analyses of mapped 27nt-RNAs on exon boundaries on coding sequence (CDS) contigs with high coverage revealed a similar mean of mapped 27nt-RNAs when compared with the whole contig. In several examples the exon boundaries were centrally arranged with respect to the matching 27nt-RNA reads (Figure 2D), which supports the idea that introns are mostly not present in the 27nt-RNA precursors. This is an argument against the hypothesis that the precursors could be long telomere-primed transcripts of whole nanochromosomes, which should include introns.

**Figure 3:**
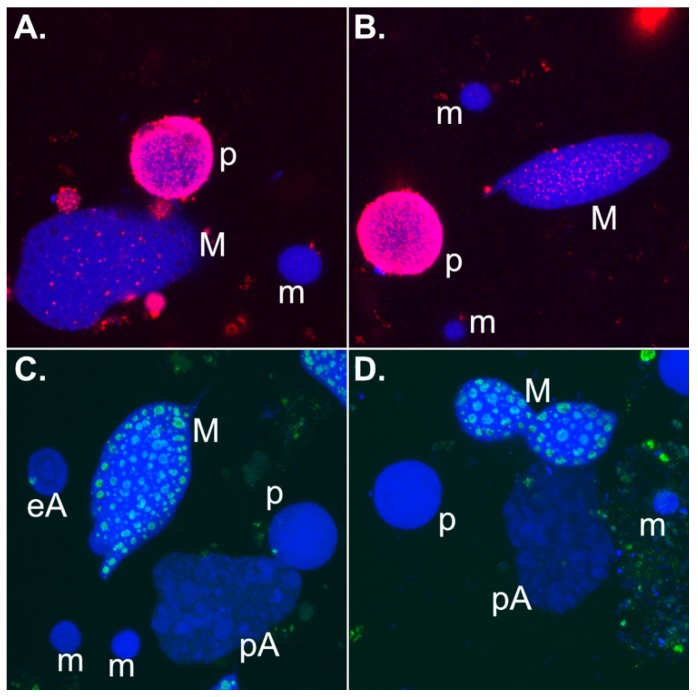
Immunolocalization of nascent RNA and the RNA processing protein fibrillarin in different nuclear types isolated from various developmental stages during *Stylonychia* sexual reproduction. False colours were assigned to each channel. Abbreviations: p: parental macronuclear fragment; m: micronucleus; M: macronucleus; eA: early macronuclear anlage; pA: polytene macronuclear anlage. (**a**,**b**) Visualization of nascent transcript using 5-FU (red signals). DNA was counterstained with To-Pro-3. (**c**,**d**) Visualization of fibrillarin using fibrillarin/Nop1p antibodies. DNA was counterstained with To-Pro-3.

Hypothetically, 27nt-RNA biogenesis might take place upon an unknown signal that stops editing of immature mRNAs, nuclear (parental/old macronuclear) export and translation of mRNAs. It seems extremely coherent that in *Stylonychia,* upon its first cellular appearance, the argonaute homolog PIWI1 that binds 27nt-RNAs and is involved in MDS protection accumulates in exactly those parental macronuclear fragments [13,24], which are the obvious nuclear types wherein 27nt-RNA biogenesis takes place and wherein the postulated mRNA substrate should be present. Moreover, upon labelling of nascent transcripts using 5-fluorouridine (5-FU) massive RNA enrichment can be visualized in parental macronuclear fragments of exconjugant *Stylonychia* (Figure 3A,B). Generally, nascent RNA in vegetative macronuclei of *Stylonychia* colocalizes with the same nuclear bodies that contain fibrillarin, which is presumably involved in rRNA processing. Therefore, there are no specialized nucleoli, which occur physically separated from other transcription sites [29,30]. In the parental macronuclear fragments fibrillarin is not detectable. Concomitantly, the former macronuclear transcription sites/fibrillarin bodies appear to vanish morphologically in parental macronuclear fragments (Figure 3C,D). This observation might be indicative for a generally stalled RNA processing in the parental macronuclei of exconjugants, and this would perfectly fit with the stop of RNA maturation proposed above, but it must be emphasized that fibrillarin can be consulted as an indirect marker at best, since it is involved in the processing rRNA but not mRNA. 

However, we have to consider that MDS protection through 27nt-RNAs can be efficient only, if all MDSs can be targeted. This would require the catholic presence of transcripts from all nanochromosomes upon initiation of 27nt-RNA biogenesis, regardless of whether they are required for vegetative growth or whether they become differentially expressed during development. How this holistic accumulation of usually differentially regulated transcripts could be achieved is unknown, but transcriptome data do indeed support this idea, since the vast majority of annotated *Stylonychia* genes is represented in the mapped RNA reads 10 and 20 hrs post conjugation ([13]; see also: http://stylo.ciliate.org/index.php/home/downloads). Conveniently, while ‘vegetative growth’ transcripts are synthesized in the non-reproductive cycle of *Stylonychia* it was observed through actinomycin D treatment experiments that RNA synthesis continues and is essential for macronuclear development for approx. 6hrs after the beginning of conjugation before it abruptly stops. Then cells become insensitive to actinomycin D treatment [31,32]. Given that their half-life is sufficiently long, the pool of ‘vegetative growth’ mRNAs could indeed be complemented by development-specific transcripts during that time window. Furthermore, the observed stop of RNA synthesis is perfectly in agreement with the postulate described above that 27nt-RNA biogenesis from mRNAs could require a checkpoint where the direction of mRNA processing changes. However, while this could lead to the simultaneous accumulation of vegetative growth transcripts and development-specific transcripts, it would remain unexplained how rarely used transcripts can become part of the ‘catholic mRNA pool’. However, a result of this putative mRNA-dependent 27nt-RNA-synthesis is that these small non-coding RNAs would occur with different coverages for different MDSs. It is completely unknown whether these differences in 27nt-RNA quantities can have influence on the efficiency of MDS protection, and it is only speculation that such efficiency difference could eventually contribute to the control of nanochromosome copy numbers in the mature macronucleus—possibly in addition to the involvement of other maternal ncRNA species as proposed previously for *Oxytricha* [9] and *Stylonychia* [8]. This speculative contribution could be suggested by the observation that there is also a positive correlation between the quantities of mRNAs, 27nt-RNAs and the nanochromosome copy numbers [13].

## 6. Conclusions

Taken together, in our review we summarize evidence that the small RNA species occurring in *Stylonychia* could result from processing of pre-mRNA or/and mRNA. With emphasis on macronuclear development it might be the case that a catholic pool of mRNAs accumulates in the parental macronuclei and does not become further edited and translated into proteins. Instead, mRNAs become converted into 27nt-RNAs, possibly involving RDRP-activity (double strand conversion, signal amplification) and Dicer-like activity (27nt-RNA processing prior to PIWI1 loading). If this proposed mechanism exists it could provide an elegant explanation of how protein-coding information (mRNA) from parental somatic nuclei (thus their transcription profiles determining the phenotype) can be converted into non-coding information that is used to transmit the functional partitioning of the parental genome to the offspring.
